# Revealing the Effect of the Molecular Weight Distribution on the Chain Diffusion and Crystallization Process under a Branched Trimodal Polyethylene System

**DOI:** 10.3390/polym16020265

**Published:** 2024-01-18

**Authors:** Min Cai, Xuelian He, Boping Liu

**Affiliations:** 1 State Key Laboratory of Chemical Engineering, East China University of Science and Technology, Meilong Road 130, Shanghai 200237, China; charminnng@126.com; 2 College of Materials and Energy, South China Agricultural University, Guangzhou 510642, China

**Keywords:** trimodal polyethylene, molecular weight distribution, short-chain branching, molecular dynamics simulation, crystallization

## Abstract

With the increasing demand for high-end materials, trimodal polyethylene (PE) has become a research hotspot in recent years due to its superior performance compared with bimodal PE. By means of molecular dynamics (MD) simulations, we aim to expound the effect of the molecular weight distribution (MWD) on the mechanism of nucleation and crystallization of trimodal PE. The crystallization rate is faster when short-chain branching is distributed on a single backbone compared to that on two backbones. In addition, as the content of high molecular weight backbone decreases, the time required for nucleation decreases, but the crystallization rate slows down. This is because low molecular weight backbones undergo intra-chain nucleation and crystallize earlier due to the high diffusion capacity, which leads to entanglement that prevents the movement of medium or high molecular weight backbones. Furthermore, crystallized short backbones hinder the movement and crystallization of other backbones. What is more, a small increase in the high molecular weight branched backbone of trimodal PE can make the crystallinity greater than that of bimodal PE, but when the content of high molecular weight backbone is too high, the crystallinity decreases instead, because the contribution of short and medium backbones to high crystallinity is greater than that of long backbones.

## 1. Introduction

Polyethylene (PE) is widely used in our daily life because of its great properties [1]. However, it is difficult for unimodal PE to achieve excellent processability and mechanical strength simultaneously. Therefore, branched high-density polyethylene (HDPE) with a multimodal molecular weight distribution has had a wide range of applications since the 1970s. In particular, the mechanical properties of multimodal PE are obviously greater than those of unimodal PE [2,3]. In general, multimodal PE consists of a high molecular weight component with great impact resistance and mechanical properties and a low molecular weight component with excellent processability and rigidity. This led to it being able to be processed as high-grade pipelines and films [4,5,6,7].

At present, bimodal PE is one of the more mature multimodal PE materials [8,9,10,11]. With the development of science and technology, the performance of bimodal PE is no longer sufficient to meet the growing demand for PE with higher performance. For PE materials, it is necessary to improve the machining performance, especially the wear resistance and crack growth resistance, which can be further improved for high-end fields such as high-strength fibers for mooring lines in the offshore oil field, protective products and high-grade gas pipe such as PE100RC, etc.

Against this background, LyondellBasell was among the first to develop a three-tank polymerization process to produce trimodal PE in 2004. Trimodal polyethylene is produced by adding ultra-high molecular weight PE to bimodal PE. It has better performance than bimodal PE and the more excellent properties required for the above high-end materials [12,13]. As a new research upsurge, the research into three-site trimodal PE catalysts at home and abroad is still at the initial stage. Based on the research basis in bimodal PE catalysts, Mülhaupt et al. [14,15,16,17] developed trimodal PE by using three different homogeneous PE catalysts to obtain Cr-Fe/tri-sites PE catalysts. And the distribution of the molecular weight can be controlled by adjusting the ratio of different catalysts. What is more, Stürzel et al. [12] used catalysts with three active sites loaded on functionalized graphene to customize the molar mass distribution of the trimodal PE to promote the self-reinforcement of PE for the production of trimodal PE. Tsou et al. [13] mixed a low amount of solution-made branching HDPE into the gas-phase-made bimodal HDPE. The trimodal PE obtained via mixing retained the great properties of bimodal HDPE with additional processability and melt strength. Eslamian et al. [18] blended 40 wt% HDPE into melt-compounded LDPE and Zeigler–Natta LLDPE, and they explored the rheological and mechanical properties of this ternary PE in detail. The conclusion was that the fine co-crystals between the LLDPE and LDPE strengthened the mechanical properties of the blend.

In order to study trimodal polyethylene, it is necessary to explore the influence of the molecular weight distribution (MWD) on the properties of it [19,20,21]. Sifri et al. [22] observed that the opposite MWD skew led to a variation in the complex viscosity and shear thinning but did not affect the strain at break. Kida et al. [23] blended a unimodal PE into a polydisperse PE to investigate the impact of PE with a high molecular weight on the mechanical properties of products. The results showed that adding the PE with the critical Mw value of 3.0 × 10^5^ could enhance the strain-hardening and strength modulus. Wu et al. [24] discussed the effect of bimodal MWD on the crystallization process and rheological and mechanical properties of PE products. It appeared that the adding of low molecular weight homo-polyethylene remarkably improved the crystallization capacity, leading to the thick lamellae and higher rapid crack propagation resistance of PE100 pipes. At the same time, the short-chain branching (SCB) characteristics also had an essential effect on the polymer products’ rheological and mechanical properties [25,26,27,28]. Mandelkern et al. [29,30,31] studied the influence of the short-chain branching concentration (SCBC) and short-chain branching length (SCBL) on the crystallization process of branched PE. In conclusion, the increasing content of SCB decreased the crystallinity of the polymer and also declined the melting temperature and wafer thickness. On the other hand, SCBL had no obvious effect on these parameters. Akpalu [32] and Gupta [33] discovered that the position of SCB changed from outside to inside the crystal region and the critical value is 10 CH_2_. Krishnaswamy et al. [34] investigated the effect of the SCB distribution on improving the mechanical properties, including the slow crack growth resistance, of PE. The results showed that distributing SCB on the higher molecular weight PE component was better.

**Figure 1:**
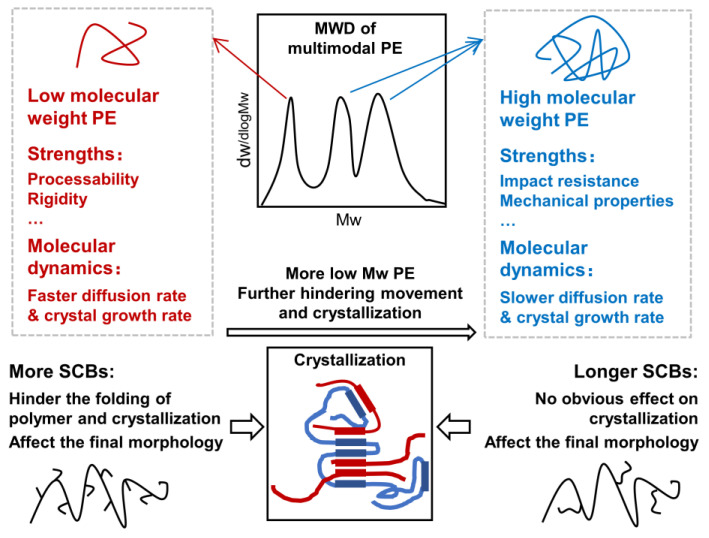
Effects of different molecular weight PE components and SCB characteristics on the properties and crystallization process of multimodal PE systems.

However, the experimental method still has some limitations, including the difficulty of imprecisely controlling the polymer microstructures and time-consuming nature. Molecular dynamics (MD) simulation, as a digital tool, is powerful when modeling the polymer chain with specify structures to study the effect of microstructures on the crystallization process of trimodal PE at the molecular level. Moyassari et al. [7] used coarse-grained MD simulations to study the influence of high molecular weight PE on the crystallinity and tie chain concentration of bimodal PE, and the increasing content of high Mw PE led to a tougher model system. Zhai et al. [35] studied the crystallization process of bimodal PE using a MD model with varying the weight fraction ration of short chains to long chains. It was found that the crystal growth rate kept decreasing with the increasing long chain content, which mainly resulted from the lower diffusion rate of chain sliding. Triandafilidi et al. [36] used MD simulations to investigate the crystallization kinetics of multimodal polymer. The results indicated that the low Mw chains were helping the high Mw chains to crystallize at the beginning. With the processing of crystallization, after all the low Mw chains had crystallized, it started to hinder and obstruct the motion and crystallization of high Mw chains. Zhao et al. [37] unraveled the effect of MWD on PE shish-kebab crystallization and discovered that wider MWD was conducive to the generation of shish nuclei and increased the rate of crystallization. What is more, the formed lamella was more regular and compact, and it had less tie chains. But the final crystallinity of the polymer had no obvious difference with the variation of the MWD. For the effect of SCB, Zhang et al. [38,39,40,41] conducted a series of MD simulation works to investigate the effects of the concentration and length of SCB on the single PE chain crystallization, and they discovered that a lower content of SCB resulted in increasing crystallization and the more regular structure of the lamellar. For the effect of SCBL, their simulation results matched well with the experimental conclusion drawn by Akpalu [32] and Gupta [33]. Choi et al. [42] also built a model of a linear low-density PE chain to discover the effect of the short-chain branching distribution (SCBD) on the final structure of PE crystals. The simulation results revealed that the crystallinity of PE with randomly distributed SCB was higher than the system with uniformly distributed SCB. In previous work, Gao et al. [43,44] explored the influence of the SCB characteristics on the crystallization process of unimodal PE and found that SCBC affected not only the crystallization kinetics but also the final morphologies, although SCBL only affected the final morphologies. By establishing a bimodal polyethylene model, Hu et al. [45,46] found that branched long backbones are more likely to produce tie chain molecules, and they elucidated the related mechanism. Unlike the short chain branches, Wang et al. [47] found and analyzed the inhibition mechanism of long chain branches on the PE crystallization by the means of MD simulation. Figure 1 provides a brief overview of the effects of different Mw PE components on the product properties, difference in molecular dynamics behavior of different Mw polymer chains, as well as the influence of the short chain branching characteristics on the crystallization process.

Most experimental or simulation research about multimodal PE mainly focuses on bimodal PE. However, as a cutting-edge material, trimodal PE has gradually entered people’s vision, and the study of the effect of MWD on the crystallization process can be helpful for the design of trimodal PE materials for industrial production. In our previous work [48], a series of trimodal PE models, including different short-chain branching characteristics, were established. The results revealed that the effect of SCBC and SCBL on the trimodal PE crystallization process was similar to that of unimodal and bimodal PE. In addition, SCBD affected the chain entanglement mechanism and caused micro phase separation. In this work, the branched trimodal PE models with different MWDs were precisely designed. The SCBC varied from 4 to 12 SCBs/1000 C, and the SCBs were distributed on the medium backbone or long backbone or both. This work explored the effect of different MWDs and microstructures on the nucleation and crystallization mechanism of trimodal PE by the means of molecular dynamics (MD) simulations. The results showed that the nucleation and crystallization mechanism of PE was different with the variation of the MWD. The concentration of the short backbone affects the form of nucleation, while the concentration of the long backbone affects the crystallization rate of the system. In addition, the response of crystallinity to SCBC and SCBD was different for trimodal PE models with different MWDs. To be specific, distributing SCBs on the long backbone or increasing the concentration of the long backbone by a small amount contributes to the improvement of crystallinity. On the contrary, the excessively high concentration of the long chain is not conducive to crystallization.

## 2. Materials and Methods

### 2.1. Models

Trimodal PE models with three different MWDs (A, B and C) were established in our work. Model A consisted of one short chain (S backbone: 1000 CH_2_), one medium chain (M backbone: 5000 CH_2_) and one long chain (L backbone: 10,000 CH_2_). Model B was made up of five S backbones, one M backbone and one L backbone. Model C was composed of ten S backbones, two M backbones and one L backbone. The SCBs were distributed equally on the M backbone (M), L backbone (L), and M and L backbones (ML). In contrast, a bimodal PE model with fifteen S backbones and three M backbones, in which the SCBs were distributed on the M backbones, was established. The mass fraction of backbones in each model is shown in Table 1, where it could be seen that the ratio of the mass fraction of the S backbone to that of the M backbone in models B and C was the same as that in the bimodal model. In addition, the branch concentration of the trimodal PE models included 4, 8, and 12 branches per 1000 carbons. Meanwhile, the branch length in the bimodal or trimodal PE models was 4C. The naming convention for the trimodal PE models identifies which backbones are inserted by branch, as well as the MWD. For instance, Tri-A-C4L4-L represents that the system consisting of one S backbone, one M backbone and one L backbone, and the SCB concentration is four branches per 1000C. At the same time, the SCBs are all distributed on the long backbone. Tri-C-C12L4-ML represents the system consisting of ten S backbones, two M backbones and one L backbone and SCBs with 4C distributed evenly on the M and L backbones with a branch concentration of 12 branches per 1000C.

### 2.2. Simulation Details

The MD simulation was operated in GROMACS. Drieding II [49] was selected as the force field of atomic interactions based on the investigation of Choi [50]. The total energy E_total_ consists of the kinetic energy E_kinetic_ and the potential energy E_potential_. The E_kinetic_ depends on the crystallization temperature. The E_potential_ includes the bond potential energy (bond-stretching, bond-bending, and torsional energies) and the nonbond potential energy (12-6 Lenard–Jones potential van der Waals energy) and could be calculated via the equation: E_potential_ = E_stretch_ + E_bend_ + E_torsion_ + E_vdW_. Integrating the motion equation within the ∆t = 2 fs via the velocity Verlet algorithm. The initial simulated box size is 500 nm × 500 nm × 500 nm. The system temperature and pressure are maintained by the Canonical Nosé–Hoover NVT ensemble and the extended Parrinello–Rahman NPT ensemble, respectively. For obtaining the equilibrium amorphous polymers, the NVT ensembles ensure that the polymer chains with trans conformations were melted at T = 800 K for 10 ns, and then the NPT ensembles ensure that the polymer chains relaxed for another 10 ns at hydrostatic pressure *p* = 0.1 MPa. After that, the trimodal PE model is quenched from 800 K to 300 K within 40 ns for crystallization process simulation. In this step, the temperature linearly decreases with time over 40 ns, and the cooling rate is 12.5 K/ns.

### 2.3. Analysis Methods

In order to accurately describe the simulation system, it is important to choose the appropriate order parameters. Depending on the actual simulation requirements, low rank or higher rank polynomials can be selected [51]. For the purpose of counting the number of orders in the trimodal PE system, the site order parameter (*SOP*) is used to calculate the local order parameter in this work [52,53,54]. Using site k as an example, the calculation of *SOP* is expressed as follows:

(1) $${SOP}_{k}=\frac{\langle 3{cos}^{2}\left(\Psi \right)-1\rangle }{2}=\frac{3}{2}{\langle \left({\vec{e}}_{ik}\cdot {\vec{e}}_{jk}\right)\rangle }_{R}-\frac{1}{2}$$

where *i* and *j* represent two vectors in a circle with a radius of 0.55 nm centered on the site, respectively, in the domain.

For a model having sites with the number of *N*, the order parameter is the average *SOP_k_* value of all the sites in the system:

(2) $$SOP=\frac{1}{N}\sum_{k=1}^{N}{SOP}_{k}$$

This equation is used to obtain the polymer crystallinity:

(3) $$X_{C}=N_{cv}/N$$

where *N_cv_* is the amount of sites with high *SOP* (greater than 0.7) and *N* is the total amount of sites in the system. *X_c_* is the fraction of sites with *SOP_k_* higher than the critical value of 0.7, which was tested by Yu et al. [54]. There would be a situation when dealing with the ordered cluster on the interfaces, where the vectors in different crystal domains paralleled to each other. So, the *SOP* of the sites where the number of orientation vectors is lower than 30, is set to 0. This means that those sites make no contribution to the calculation of the average *SOP*. The angle and distance between the crystalline chain segments are considered to determine whether the crystalline chain segments are in the same crystalline region [55,56]. In our work, if the atom distance is close enough (lower than 0.68 nm), with a high average cosine value between two orientation vectors (greater than 0.82), it could be argued that those vectors are enclosed by the same crystalline or nucleus region in the ordered regions with an *SOP_k_* higher than 0.7 [57,58]. The clusters with more than 8 CH_2_ unites are defined as nuclei or crystal domains.

The trans state population (*Pi*) is obtained by dividing the amount of methylene units with dihedral angles in the range of 180° ± 15° by the total amount of methylene units with different trans dihedral angle ∑*N_i_* in the whole system,

(4) Pi=Ni/∑Ni

The mean square displacement (*MSD*) is a parameter that represents the deviation distance of all the atoms in the box and can be used to compare the mobility of polymer chains. It is calculated using the following equation [59],

(5) $$MSD(t)=\frac{1}{N}\sum_{i=1}^{N}{\left[r_{i}\left(t\right)-r_{i}\left(0\right)\right]}^{2}$$

where *r_i_* represents the positional coordinate of atom *i*, 0 denotes the initial time and *t* denotes time *t*.

## 3. Results

### 3.1. Mobility of Backbones in the Nucleation and Crystal Growth Steps

**Figure 2:**
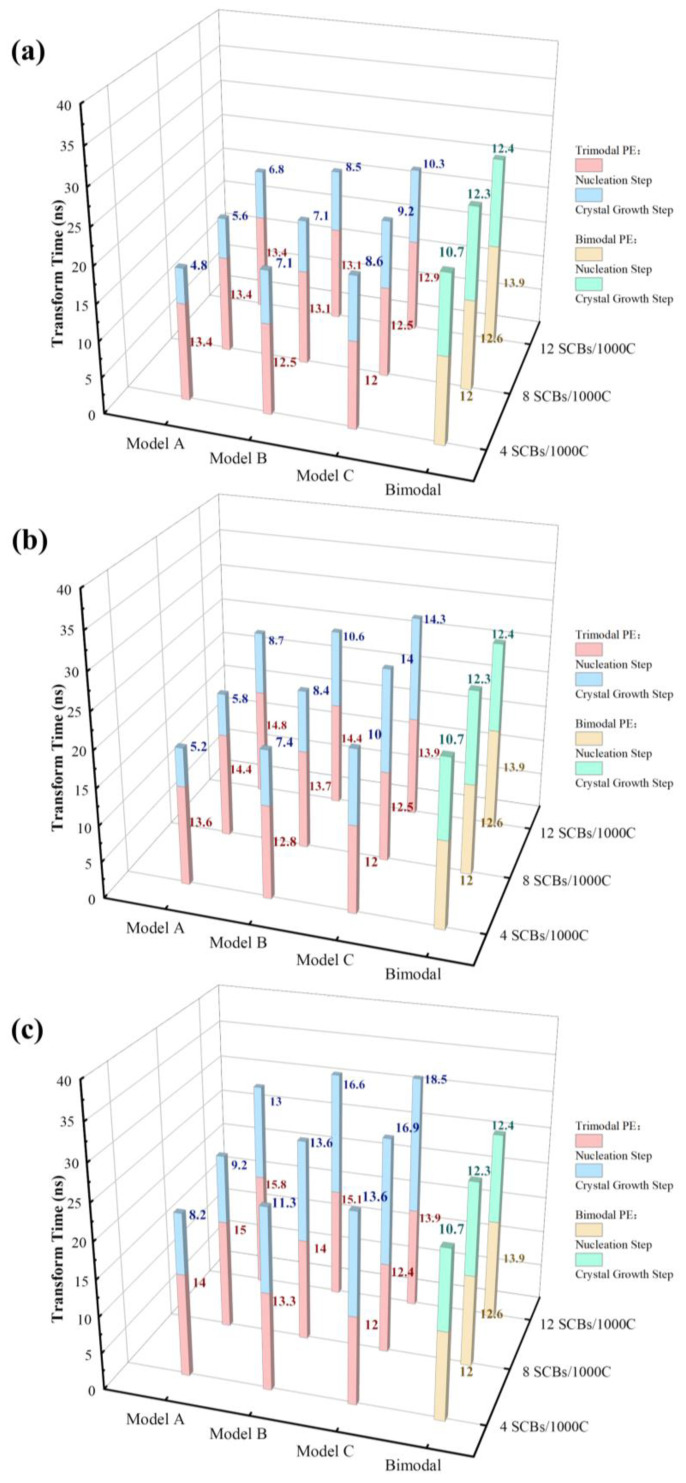
The variation in the transform time at the nucleation step and crystal growth step with different MWDs and SCBs distributed on the (**a**): M backbones; (**b**): L backbones; and (**c**) M and L backbones.

Looking at the big picture, the variation in the transform time at the nucleation stage and crystal growth stage with different MWDs and SCBDs are illustrated in Figure 2, and the transform time represents the time it takes for each stage to go from start to finish. The nucleation step was defined as the process at the initial moment when the trans state population of the system increased slowly. In this process, the nucleation precursor was continuously formed and abled, and the crystallinity did not change significantly. After that, the trans state population increased rapidly until the moment when the trans state population did not change, which was called the crystal growth step. During this process, due to the formation of stable crystal nuclei, the polymer chains began to fold and form lamellae, resulting in a rapid increase in the crystallinity of the system. The comparison between trans state population of each model will be described in detail later. It was not difficult to find that when the concentration, length and distribution of the SCBs were completely the same, the model with a lower content of high molecular weight backbones (like model C) seemed to have a faster nucleation rate than the model with a higher content of high molecular weight backbones (like model A), but it needed a longer time to reach a stable state of crystallization. At the same time, the bimodal PE model also had a nucleation rate similar to model C, and this model also needed more time for crystallization. It could be seen that with the increase in the high molecular weight backbone concentration, the nucleation step of the system became slower and slower. In addition, the crystal growth step of the trimodal PE model was generally faster than that of the bimodal PE model when the SCBs were distributed only on the medium or long backbones, while the crystallization rate of the trimodal PE model was greatly slowed down, even less than that of bimodal PE model in some cases, when the SCBs were distributed on both the medium and long backbones.

The phenomenon that the transformation time of the crystal growth step varied with the MWD was actually easy to explain. On the one hand, with the increasing content of short backbones, the methylene sequence length (MSL) relationship of each model was model A > model B > model C. However, a smaller MSL was not conducive to chain folding and flipping, leading to a decrease in the crystallization rate. On the other hand, a system with a higher content of short backbones would inevitably have a larger number of terminal groups and more space available for the free movement of polymer backbones, which resulted in stronger diffusion and entanglement between the backbones and further hindered the movement and folding of all the backbones. Under the joint action of these two factors, the transformation time of the crystal growth step presented the rule shown above. Moreover, when the SCBs were distributed on both the medium and long backbones, the transformation time of the crystal growth step was generally greater than that of the bimodal PE model because most of the backbones were affected by the effect of the SCBs on their motion ability.

**Figure 3:**
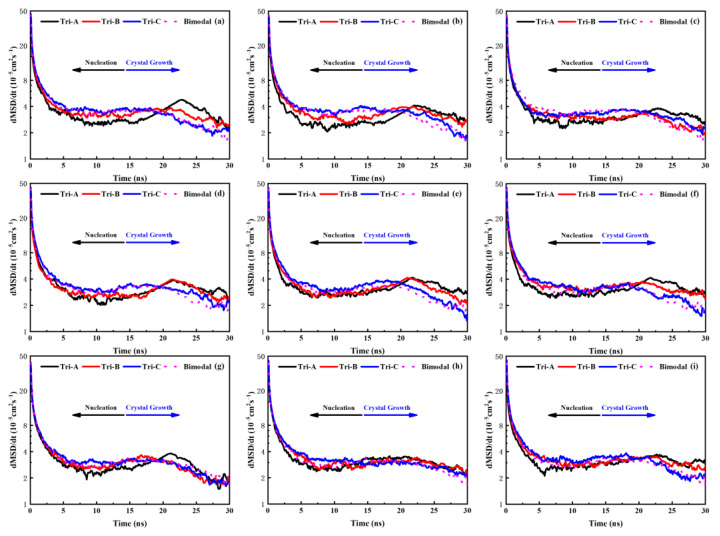
The mobility of the backbones under different MWDs and SCBCs: (**a**) C4L4-M; (**b**) C4L4-L; (**c**) C4L4-ML; (**d**) C8L4-M; (**e**) C8L4-L; (**f**) C8L4-ML; (**g**) C12L4-M; (**h**) C12L4-L; and (**i**) C12L4-ML.

**Figure 4:**
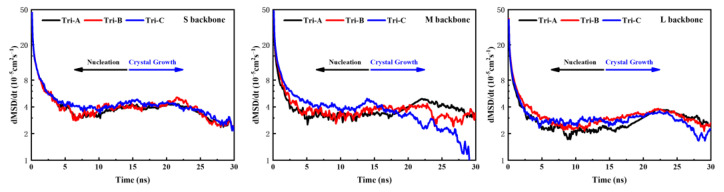
The mobility of backbones with different molecular weights under different MWDs in the C4L4-L system.

To better prove this point, Figure 3 provides the change in the dMSD (dt = 0.1 ns) over time to express the diffusion capacity or mobility of the backbones in the system under the same SCB characteristics but different MWDs. It is apparent from this figure that the variation in the mobility of the backbone with the time of the different models was complementary to the law of the transformation time of the nucleation step and crystal growth step discussed above. That is, the mobility of the backbone of the model with a lower short backbone concentration (like model A) was weaker in the nucleation stage, while the mobility of the backbone became stronger in the crystallization process. On the contrary, the mobility of the backbone of the model with a higher short backbone concentration (like the bimodal model and model C) was faster in the nucleation stage, but the mobility of the backbone continued to drop during the crystallization process. Due to the stronger diffusion capacity of short backbones, there were good reasons to believe that the diffusion of the short backbones mainly occurred in the nucleation stage, considering the variation in the dMSD/dt with time in the different PE models. Therefore, it also offered us a hint that a great deal of short backbones as an obstruction hindered the diffusion of the medium and long chains at the crystal growth stage in the model with a higher concentration of short backbones. Take model C4L4-L as an example: Figure 4 shows the mobility of the S, M and L backbones in trimodal PE under different MWD systems. It can be noted that the movement of the short backbone in the three trimodal PE models was very similar, and the dMSD/dt of the S backbone at the nucleation stage was significantly larger than that of other molecular weight backbones. For the medium and long backbones, the differences in their mobility between the three trimodal PE models can be clearly observed (the difference between L backbones is not obvious because there are SCBs distributed on it, which reduces the motility of L backbone). At the nucleation stage, the motility of the medium and long backbones is: model C > model B > model A, and it is completely the opposite at the crystal growth stage. This proves that in systems with a higher content of S backbones, the short backbones will hinder the motility and diffusion of the medium and long backbones during the crystal growth stage, thereby affecting the crystallization process of the trimodal PE system.

However, a smaller MSL would not only slow the crystallization rate but in theory would also slow the nucleation rate, because SCB, as a defect, prevented the enrichment of trans carbon bonds. In addition, the backbones with a higher molecular weight contributed more to the nucleation process, because the short backbones were difficult to fold into nuclei within the chain. Therefore, the model with the lower concentration of SCBs (like model A) should nucleate faster. But, as shown in Figure 2, the transform time of the nucleation step shows the opposite pattern. In conclusion, it was reasonable to believe that the mechanism of nucleation changed as the concentration of short chains decreased.

### 3.2. Mechanism of Nucleation and Crystallization

**Figure 5:**
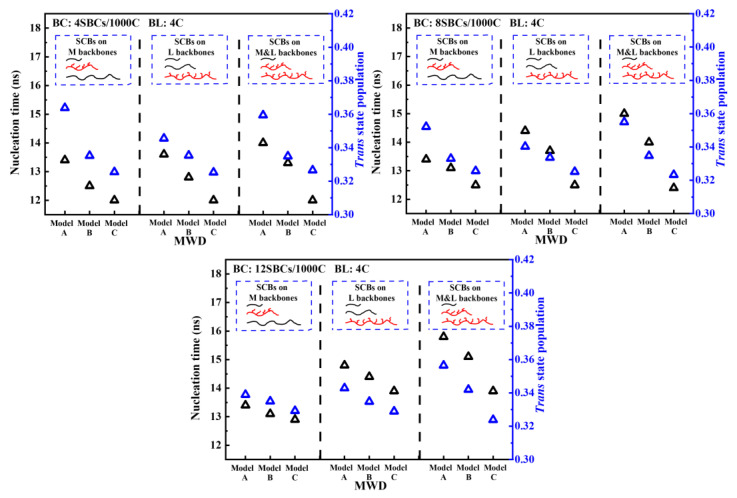
The variation in the transform time and trans state population at the nucleation stage with different MWDs and SCBDs (black triangle: nucleation time; blue triangle: trans state population).

The transformation time and trans state population corresponding to the nucleation stage of the trimodal PE with different MWD are shown in Figure 5. Previously, it was observed in Figure 2 that models with a higher long backbone content had a shorter nucleation time. From Figure 5, it can be seen that the models with a lower long backbone content also had a lower trans state population at the end of the nucleation stage. This meant that the nucleation capacity was weakened in the nucleation stage when the long backbone content decreased. But that still could not explain why the trans state population of the models with a lower long backbone concentration started to increase substantially earlier. That is, the apparent nucleation time was shorter.

To explain this phenomenon, it was necessary to explore the ways of nucleation and crystallization. In general, there were two main ways of nucleation and crystallization [60,61]. One was the way of inter-chain, which crystallized mainly via the formation of stretching chains, and the other was the way of intra-chain, which crystallized mainly via the formation of folding chains. Intra-chain nucleation required giving the chain more space to fold than inter-chain nucleation. For the model with more short backbones, there were more terminal groups in the system, and it was easier to generate free movement space between the backbones. Moreover, due to the stronger motion ability of the short backbones, the degree of entanglement of the system was more intense. In the nucleation process, for the model with a lower concentration of short backbones, without the restriction of the short backbones, the medium and long backbones, which diffuse more slowly, were more likely to fold and intra-chain nucleate, resulting in larger and more stable nuclei, and apparently exhibiting a higher trans state population. In a model with a higher concentration of short backbones, the short backbones diffused and tangled rapidly, which made the short backbones in the system restrict the folding of each backbone and be more inclined to inter-chain nucleation, resulting in a lower trans state population. Inter-chain nucleation of the short backbones led to the formation of smaller lamellae early in the crystallization process, while the medium and long backbones were still diffusing and folding for the nucleation due to the obstruction of the short backbones. As a result, a “strange” phenomenon was observed, namely that the system like model C had a faster nucleation step but slower crystal growth step.

**Figure 6:**
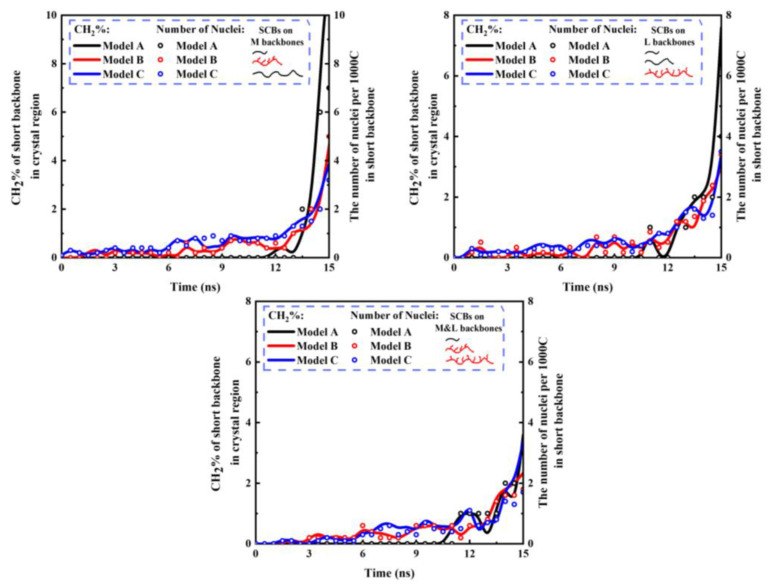
CH_2_% of the short backbone in the crystal region and the number of nuclei per 1000C of the short backbone in the trimodal PE with different MWDs under 4SCBs/1000C and 4C for SCBL.

**Figure 7:**
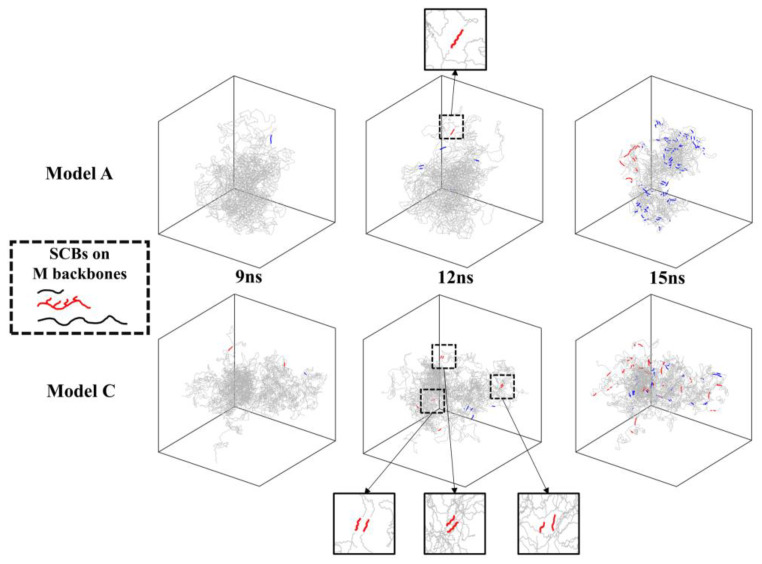
Comparison of the crystal regions of the backbones with different molecular weights between model A and model C at different times under C4L4 (red: crystalline region of short backbones; blue: crystalline region of medium and long backbones; gray: amorphous region).

To verify this hypothesis, the CH_2_% in the crystal region and the number of nuclei per 1000C of the short backbone in the trimodal PE model with different MWDs are illustrated in Figure 6. It can be observed that the short backbones in model C had already started to nucleate and partially crystallize in the early nucleation stage, and this phenomenon also occurred in model B. Figure 7 shows the crystallization process of different backbones of the trimodal PE model with different MWDs at the nucleation stage and the beginning of the crystal growth stage when the SCBs were distributed on the medium backbone (more comparison results under different SCBD are provided in the Supplementary Material as Figure S1). It can be observed that at 9 s, there was no obvious difference between the two models except that model C had a few short backbones that had formed few nuclei. At 12 s, the nucleation of the medium and long backbones in the two models was basically the same. However, the short backbone of model A had only one nucleus, which only can be formed by the intra-chain nucleation, while the short backbones of model C had 6–8 nuclei formed by the inter-chain nucleation (the two nuclei are close to each other and parallel). This indicated that inter-chain nucleation was easier to occur in model C, which had a higher concentration of short backbones. Because the diffusion capacity of the short backbone was greater, the nucleated short backbone would quickly begin to crystallize and occupy the crystallization space of the medium and long backbones. From the morphologies of 15 s, it can be seen that the nuclei formed by the short backbone in model A only distributed in a small part of the system, and a large number of CH_2_ units in the medium and long backbones had begun to crystallize. However, the nuclei formed by the short backbones in model C were distributed in various positions, even in the central region of the crystal, and the number of CH_2_ units in the medium and long backbones was significantly less than that in model A. Since the space was occupied by the short backbones, the medium and long backbones with poor diffusion capacity in model C took longer to fold and crystallize to avoid the crystallized short backbones.

**Figure 8:**
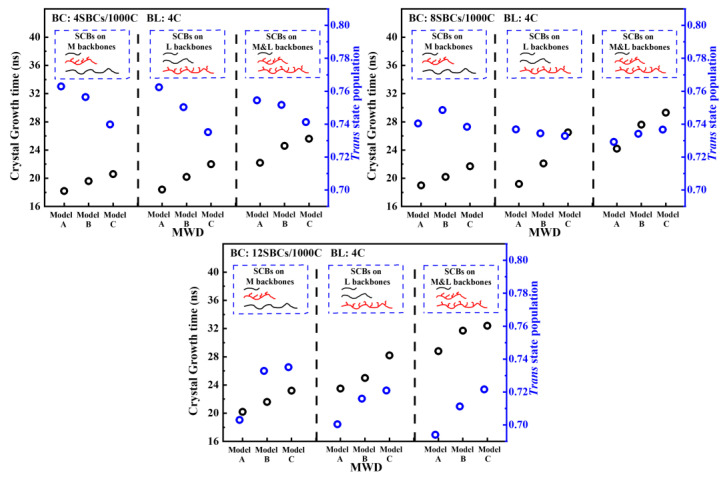
The variation in the crystal growth time and trans state population at the crystal growth stage with different MWDs and SCBDs.

**Figure 9:**
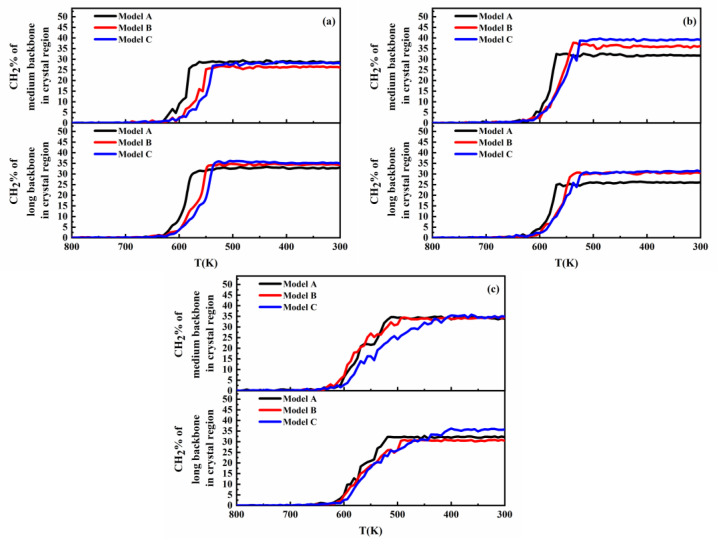
CH_2_% of the medium backbone and long backbone in the crystal region with different MWDs in the trimodal PE model under (**a**) C4L4-M; (**b**) C4L4-L; and (**c**) C4L4-ML.

**Figure 10:**
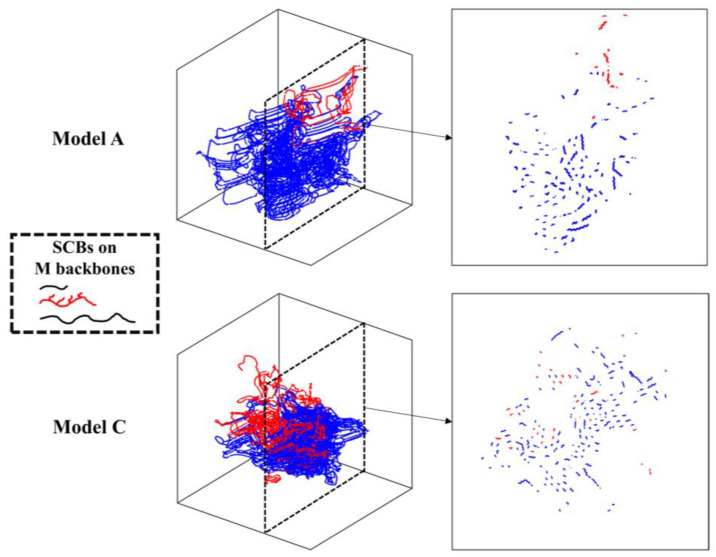
The final morphology and section diagram of model A and model C under C4L4 (red: short backbones; blue: medium and long backbones).

Figure 8 shows the variation in the crystal growth time and trans state population at the crystal growth stage with different MWDs and SCBDs. In addition to the obvious observation that the model with a lower long backbone concentration had the slowest crystal growth time, it can also be observed that the trans state population of the trimodal PE decreased with the increase in the SCB concentration, but the decreasing rate was model A > model B > model C. This was mainly due to the fact that the crystallization capacity of the PE chain with SCBs was significantly decreased with the increase in the SCB concentration, but the crystallization capacity of the short backbone was not significantly affected. Therefore, model C, with the lowest concentration of short backbones, was the least affected, while model A, with a high concentration of medium and long backbones, would be the most affected. Returning to the discussion of the crystal growth time, Figure 9 shows the variation in the CH_2_% in the medium and long backbones in the crystal regions with the time in the trimodal PE model with different MWDs. It can be noticed that the medium and long backbones in model C, with a lower long backbone concentration and higher short backbone concentration, took longer time to reach the stable state of crystallization. Meanwhile, by studying the final morphology of model A and model C when the SCBs were distributed on the medium backbone as shown in Figure 10 (more comparison results under different SCBDs are provided in the Supplementary Material as Figure S2), it is clear that the short backbone of model A occupied only a small part of the crystal region, while the short backbones of model C were entangled and crystallized with the medium and long backbones everywhere. This phenomenon could be seen more clearly by observing the section diagram. There was no short backbone between the medium and the long backbones in most of the crystal regions in model A. On the contrary, the segments of the three different molecular weight backbones in model C were evenly distributed in the crystal region. This indicated that the difference in the crystal growth stage was also caused by the diffusibility of the trimodal PE models with different MWDs. For the model with a lower concentration of short backbones, after nucleation, the diffusion of each backbone was not affected due to the small number of backbones in the system, so that the faster folding and formation of lamella could be achieved. However, for the model with a higher concentration of short backbones, the backbones were entangled with each other, and the diffusion capacity of the backbones, especially the medium and long backbones, was greatly affected by the crystallized short backbones, thus increasing the time required for crystal growth (which has been elaborated in the discussion of Figure 4 in Section 3.1).

**Figure 11:**
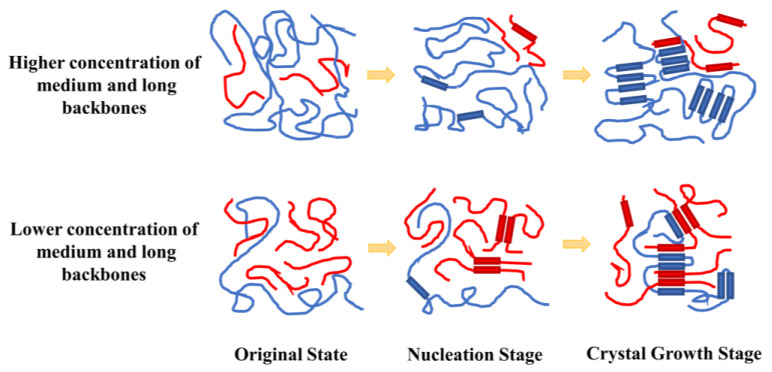
Schematic illustrations of the mechanism of the nucleation and crystallization under different MWDs (blue line: medium and long backbones in the amorphous region; red line: short backbones in the amorphous region; blue block: medium and long backbones in the crystalline region; red block: short backbones in the crystalline region).

To make it easier to understand, the nucleation and crystallization mechanism are depicted in Figure 11. For the model with a small amount of high molecular weight backbone, the higher concentration of the short backbones would tend to nucleate inter-chain and start to partly crystallize at the early nucleation stage because the shorter backbones with great motion ability increased the degree of entanglement that prevented nucleation and crystallization by means of folding, which led to an early increase in the trans state population. This made it appear that the nucleation process had been completed at this point. However, at this time, the intra-chain nucleation process of the long backbone had not been completely completed, that is to say, the crystal growth stage was actually a mixing process of the crystallization of the short backbones and the nucleation and crystallization of the medium and long backbones. The nucleation time is not the actual time required for nucleation to be fully completed. And some of the crystallized short backbones hindered the folding and crystallization of the medium and long backbones, further reducing the diffusion capacity of the system and delaying the time required for crystal growth. On the other hand, this was difficult to achieve in the model with a lower short backbone concentration. Because there were fewer backbones in the system, the diffusion of each backbone was not impeded, making it easier for the backbones to nucleate and crystallize through inter-chain folding. The formation of nuclei depended mainly on the folding of the long backbone, which was less diffusible, so its nucleation time represented the true nucleation time. In this model, the short backbones could only be crystallized based on the nuclei formed by the long backbones dispersedly after the nucleation process of the medium and long backbones was completed. As a result, the crystallization of the short backbone cannot intervene in the diffusion and crystallization of the medium and long backbones in a large range. This made the crystallization process smoother and took less time.

### 3.3. Crystallinity

**Figure 12:**
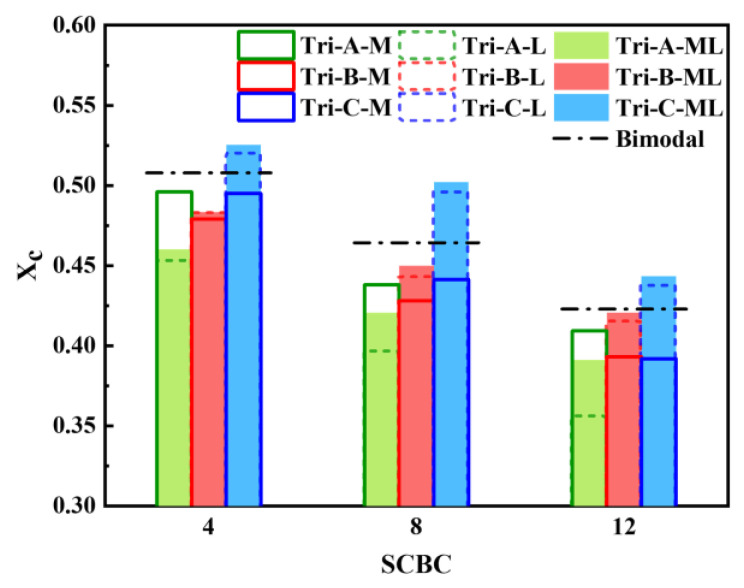
*Xc* of different trimodal and bimodal PE models at different SCBCs with SCBs distributed on the M backbone, L backbone, M and L backbones.

As an important factor affecting the polymer properties, the *Xc* of each model under different SCBCs and SCBDs is shown in Figure 12. The solid line columns in Figure 12 compare the trimodal PE models with all the SCBs distributed on the M backbone, and it was not difficult to find out that the *Xc* of all the trimodal PE models was significantly lower than that of the bimodal PE model (black dotted line). This was because the mass fraction of the M backbone of the bimodal polyethylene model was the largest, which means that the MSL of the bimodal PE model was larger than all the trimodal PE models when the SCBC was the constant. This ensured that the M backbone in the bimodal PE model was easier to fold and crystallize. On the other hand, the short backbone was beneficial to a high *Xc* [62,63]. And the short backbone content of the trimodal polyethylene models was lower than that of the bimodal model, which weakened the crystallization ability of the trimodal PE models. At the same time, this could also explain why the trans state population of model A, with the lowest short backbone concentration in Figure 8, decreased significantly with the increase in the SCBC. Combining these two factors, the *Xc* of the trimodal PE model with SCBs distributed on the M backbone was smaller than that of the bimodal PE model.

The dotted line columns in Figure 12 illustrate the *Xc* of different models with all the SCBs distributed on the L backbone, which showed a completely different phenomenon than when the SCBs were distributed on the M backbone. It was not difficult to see that the *Xc* of model C was obviously greater than that of the bimodal model, while the *Xc* of models A and B was still less than that of the bimodal model, and the *Xc* of model A was the smallest. In addition, compared with the case of the SCBs distributed on the M backbone, the crystallization capacity of models tri-B-L and tri-C-L was stronger than that of models tri-B-M and tri-C-M, while the crystallization capacity of model tri-A-L was weaker than that of model tri-A-M. For model A, the mass fraction of the short chain was too low, so that the *Xc* was mainly contributed by the medium backbone and the long backbone. When the SCBs were distributed on the long backbone, the crystallization process of most of the carbon chains in the system was seriously affected, resulting in the decline in the *Xc*. For model B and model C, the mass fraction of both the short backbone and medium backbone in the system was 50% and 75%, respectively. These carbon chains with strong crystallization ability were not affected by the SCBs, so the *Xc* of these models was improved to different degrees. More specifically, when the concentration of the long backbone was low (such as model C), the distribution of the SCBs on the long backbone released the strong crystallization capacity of a high concentration of short and medium backbones, making it show a higher *Xc* than bimodal PE. However, with the continuous increase in the long backbone concentration, it also meant a reduction in the short and medium backbone concentration, which was not conducive to a high *Xc*, so the *Xc* continued to decrease with the increase in the long backbone concentration and quickly dropped below the *Xc* of the bimodal PE.

In addition, it can be seen from the packed columns in Figure 12 that the change law of crystallinity with different SCBCs when the SCBs were evenly distributed on the medium and long backbones was basically the same as that when the SCBs were distributed on the long backbone. In addition, the *Xc* of each trimodal PE model with SCBs distributed on both the medium and long backbones was slightly higher than that of the model with SCBs distributed on the long backbone. This is because, although the SCBs were distributed on both the medium backbone and the long backbone, which reduced the crystallization capacity of these two kinds of backbones, the MSL of the system was greatly increased, which was conducive to the improvement in the *Xc*. It could be inferred that the two competing effects were that the improvement of MSL dominated and eventually lead to a small increase in the *Xc*.

Finally, the results of the investigation into the crystallization mechanism showed that the *Xc* of the trimodal PE was mainly determined by the MSL, SCBD and high molecular weight backbone concentration. The effect of the MSL or SCBC on the crystallization process of the trimodal PE was similar to that of the unimodal or bimodal PE, and smaller MSL tended to form crystals with a lower *Xc*. The influences of the SCBD and high molecular weight backbone concentration on the crystallization process of trimodal PE were complementary. When the SCBs were distributed on the medium backbone, the addition of a high molecular weight backbone had no benefit to the increase in the *Xc*. When all or part of the SCBs were distributed on the long backbone, adding a small amount of high molecular weight backbone was conducive to the improvement of the *Xc* of the system, while adding too much high molecular weight backbone would decrease the *Xc* instead.

## 4. Discussion

In our work, a series of trimodal PE models with different MWDs and SCB characteristics were established. Molecular dynamics simulation was conducted to study the molecular mechanism and effect of the MWD, especially the mechanism of nucleation and crystallization of the trimodal PE at the molecular level.

When the SCB was only distributed on only one kind of backbone, the crystallization rate of trimodal PE was faster than that of bimodal PE, because the chain entanglement caused by the high content of low molecular weight PE hindered the movement and folding of the other backbones and thus reduced the crystallization rate. However, the situation was quite the opposite when the SCB was distributed on the two kinds of components with different molecular weight, because the SCB greatly reduced the motility of the branched polymer chains.

In addition, the variation in the MWD also affected the nucleation and crystallization mechanism of PE. When the low molecular weight component content of trimodal PE was low, the nucleation of the PE mainly depended on the intra-chain nucleation of the high molecular weight PE. The high molecular weight component was conducive to the nucleation process but had weaker chain movement ability, so it took longer to nucleate but had more nuclei to speed up the rate of crystallization. When the content of the low molecular weight PE was high, inter-chain nucleation occurred because the low molecular weight components had greater motion ability and became tangled up with each other, leading to gradually less space for the high molecular weight polymer chains to fold and nucleate. In terms of the trans state population, the nucleation step was faster, but the crystallized low molecular weight PE chains hindered the movement and folding of medium and high molecular weight PE chains, making the crystallization process take a longer time.

In terms of crystallinity, when the SCBs were distributed on the medium molecular weight PE chains, the crystallinity of trimodal PE was lower than that of bimodal PE, because the high molecular weight polymer chains were not conducive to the improvement of crystallinity. However, when the SCBs were distributed on high molecular weight PE chains or both medium and high molecular weight PE chains, if the content of high molecular weight component was low, the crystallinity of trimodal PE would increase and exceed that of bimodal PE. This was because the medium molecular weight PE chains with stronger crystallinity ability were affected by less SCBs, which was conducive to the improvement of crystallinity. However, when the mass fraction of the high molecular weight PE chains continued to rise (from 33.34% to 62.5%), the content of low and medium molecular weight PE chains also continued to decrease (from 66.66% to 37.5%), which led to the continuous decline of the crystallinity, even lower than that of the bimodal PE.

In conclusion, the composition of different molecular weight PE chains in a trimodal PE system affected the crystallization process and properties of the PE. The model established in this paper could provide help and guidance for designing the microstructure of trimodal polyethylene to a certain extent.

## Figures and Tables

**Table 1:** Mass fraction of backbones with different molecular weights in different MWDs.

	ω (S Backbone)	ω (M Backbone)	ω (L Backbone)
Model A	6.25%	31.25%	62.5%
Model B	25%	25%	50%
Model C	33.33%	33.33%	33.34%
Bimodal	50%	50%	0%

## Data Availability

The raw data supporting the conclusions of this article will be made available by the authors on request.

## Supplementary Materials

The following supporting information can be downloaded at https://www.mdpi.com/article/10.3390/polym16020265/s1, Figure S1: Comparison of the crystal regions of the backbones with different molecular weights between model A and model C at different times under C4L4 and SCBs on (a): M backbones; (b) L backbones; and (c): M and L backbones (red: crystalline region of short backbones; blue: crystalline region of medium and long backbones; gray: amorphous region); Figure S2: The final morphology and section diagram of model A and model C under C4L4 and SCBs on (a): M backbones; (b) L backbones; and (c): M and L backbones (red: short backbones; blue: medium and long backbones).
