# Supplementary material for: Revealing the Effect of the Molecular Weight Distribution on the Chain Diffusion and Crystallization Process under a Branched Trimodal Polyethylene System
Source: Polymers (Basel). 2024 Jan 18;16(2):265. doi: 10.3390/polym16020265 (PMC10818820; doi:10.3390/polym16020265)
Supplement: Supplementary file 1 [file polymers-16-00265-s001.zip › polymers-2806444-supplementary.pdf]

polymers

Supplementary Materials

Revealing the Effect of the Molecular Weight Distribution on the Chain Diffusion and Crystallization Process under a Branched Trimodal Polyethylene System

Min Cai [1], Xuelian He [1,*] and Boping Liu [2,*]

[1] State Key Laboratory of Chemical Engineering, East China University of Science and Technology, Meilong Road 130, Shanghai 200237, China; charminnng@126.com
[2] College of Materials and Energy, South China Agricultural University, Guangzhou 510642, China
* Correspondence: hexl@ecust.edu.cn (X.H.); boping@scau.edu.cn (B.L.); Tel.: +86-021-64253364 (X.H.)

This document includes Figure S1 and Figure S2 mentioned in the article:

Figure S1 compare the crystal regions of the backbones with different molecular weights between Model A and Model C at different time under C4L4 and different short chain branching distribution.

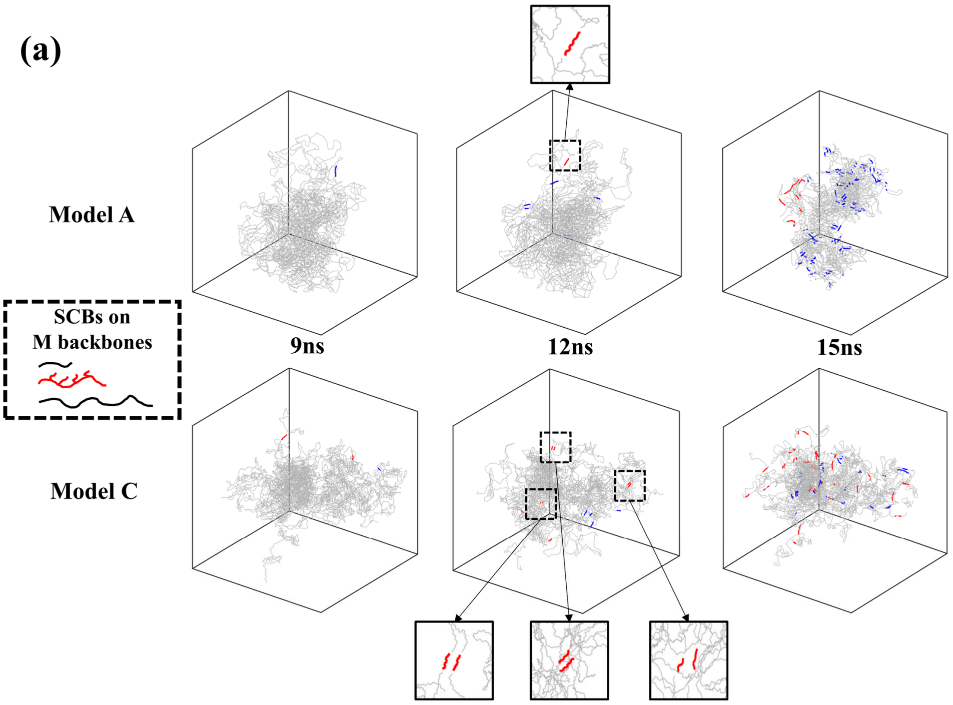

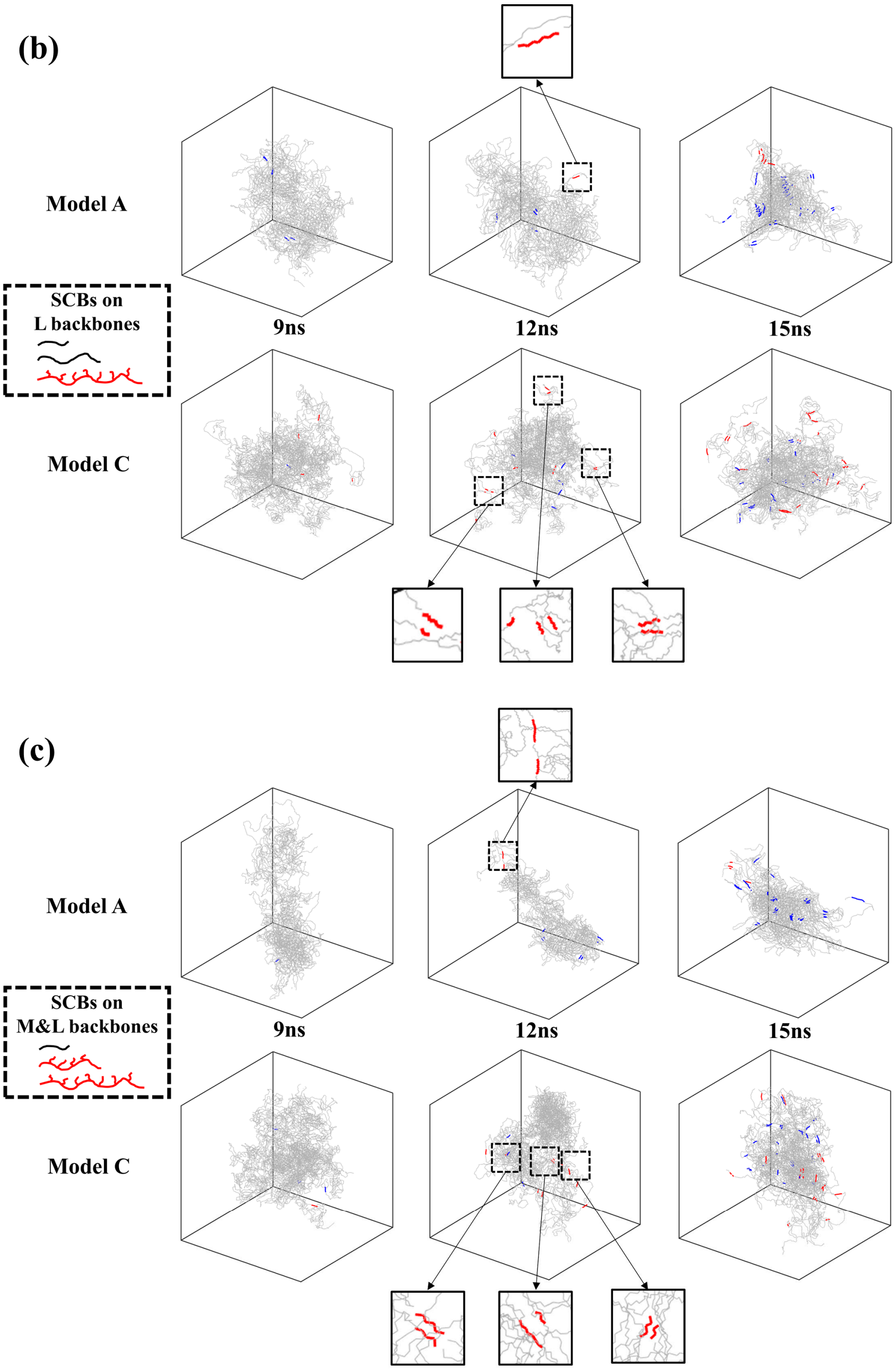

Figure S1. Comparison of crystal regions of the backbones with different molecular weights between Model A and Model C at different time under C4L4 and SCBs on (a): M backbones; (b) L backbones; (c): M & L backbones (red: crystalline region of short backbones; blue: crystalline region of medium and long backbones; grey: amorphous region)

Figure S2 shows the final morphology and section diagram of Model A and Model C under C4L4 and different short chain branching distribution.

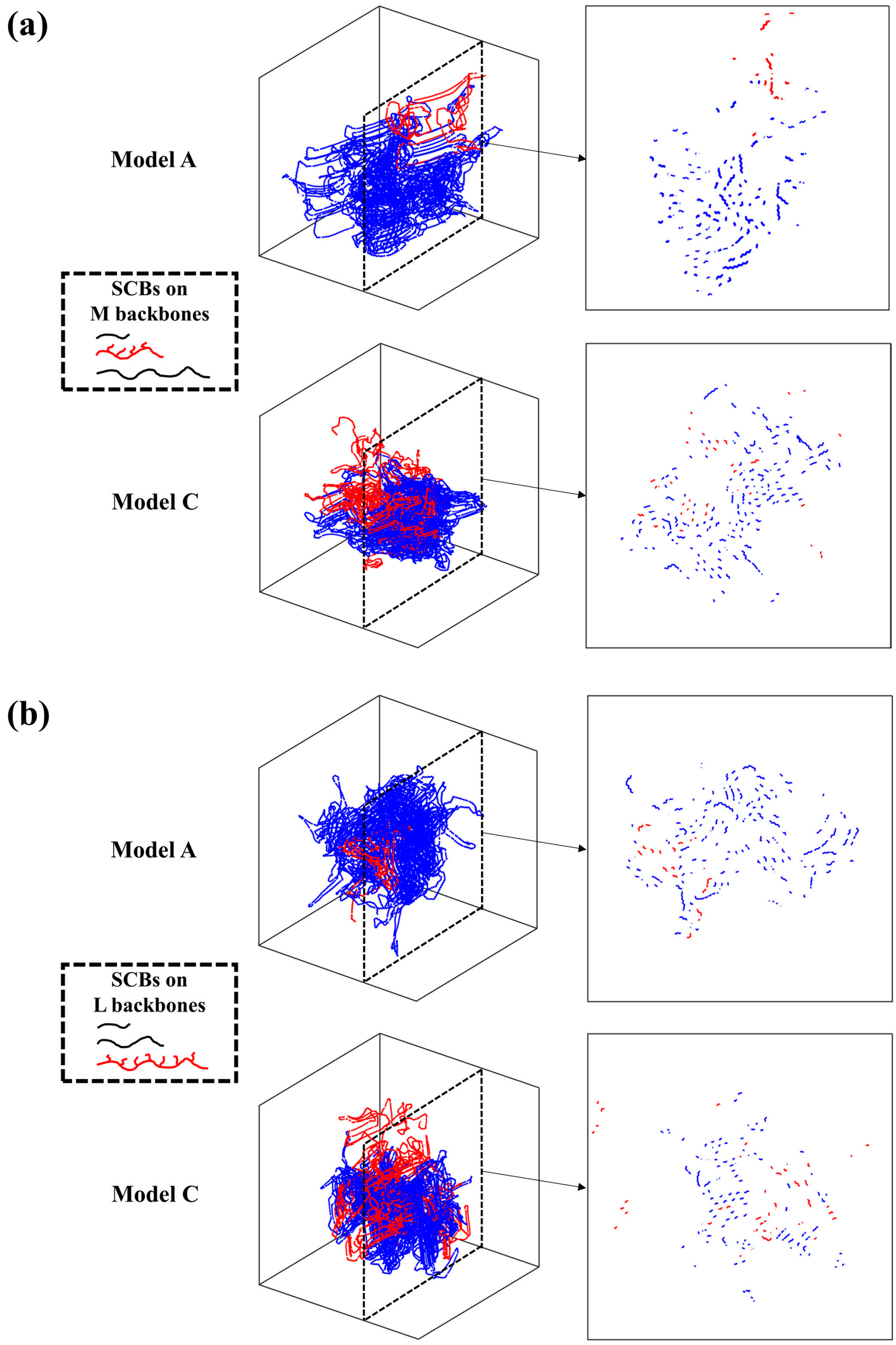

(a)
Model A
SCBs on
M backbones
Model C
(b)
Model A
SCBs on
L backbones
Model C

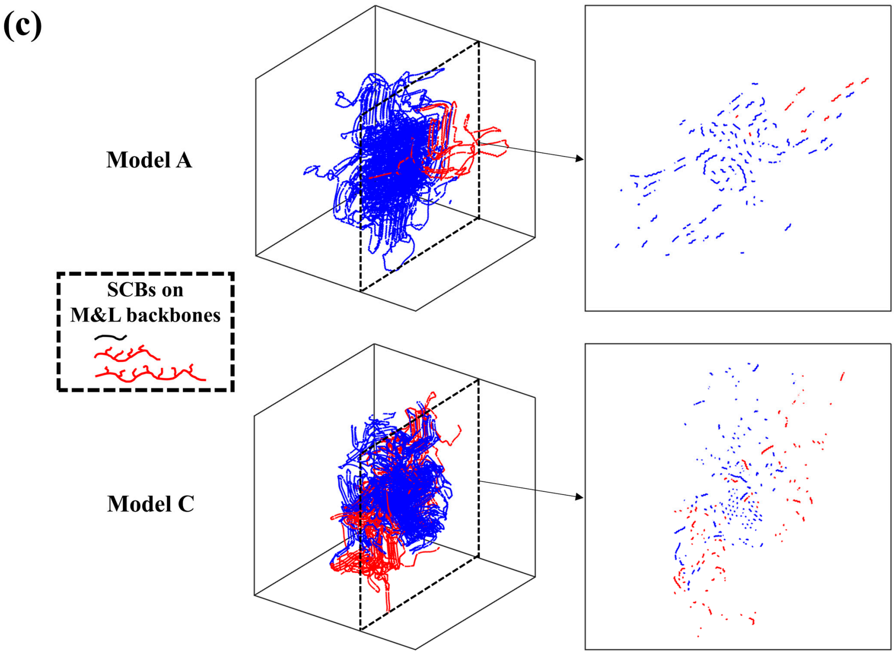

Figure S2. The final morphology and section diagram of Model A and Model C under C4L4 and SCBs on (a): M backbones; (b) L backbones; (c): M & L backbones (red: short backbones; blue: medium and long backbones)
